# Frontoparietal oscillatory dynamics support the development of fluid reasoning in children and adolescents

**DOI:** 10.1162/IMAG.a.1133

**Published:** 2026-02-09

**Authors:** Sarah L. Greenwood, Haley R. Pulliam, Monica N. Clarke-Smith, Saige C. Rasmussen, Grace E. Parolek, OgheneTejiri V. Smith, Brittany K. Taylor

**Affiliations:** Institute for Human Neuroscience, Boys Town National Research Hospital, Boys Town, NE, United States; Center for Pediatric Brain Health, Boys Town National Research Hospital, Boys Town, NE, United States; Department of Pharmacology and Neuroscience, Creighton University, Omaha, NE, United States

**Keywords:** neurodevelopment, high-order cognition, oscillations, sensitive period, gamma, P-FIT

## Abstract

The neural basis of fluid intelligence (*Gf*) has been ascribed to a distinct network of parietal and frontal brain regions referred to as the P-FIT (parieto-frontal integration theory of intelligence) network. These neural substrates rapidly mature during adolescence, although the maturation of the oscillatory dynamics serving *Gf* has seldom been studied. Therefore, the present study utilized the largest sample to date to investigate the development of these neural dynamics in 104 youth ages 8 to 15 years old who completed an abstract reasoning task during magnetoencephalography (MEG), and behavioral matrix reasoning and vocabulary assessments. We detected multispectral age- and sex-related sensitivity across the P-FIT network, including oscillatory responses within the superior temporal gyrus, dorsolateral prefrontal cortex, and inferior frontal gyrus. Perhaps most notably, we found that changes in neural dynamics within the bilateral superior parietal lobules (βs = .157 to -.126) and right lingual gyrus (β = .068) significantly mediated age-related improvements in *Gf* abilities. These large-sample findings are consistent with the P-FIT model of fluid intelligence and serve to elucidate the neural dynamics supporting *Gf* development.

## 1 Introduction

The development of fluid intelligence (*Gf*) is broadly important for higher-order cognition, analogical reasoning, and problem solving (Dix et al., 2016). *Gf* development coincides with the maturation of numerous cognitive abilities during the transition into adolescence, which is a highly dynamic period of development (Larsen & Luna, 2018). Crucially, the maturation of *Gf* is consistently linked to academic performance among adolescents, which highlights its significance during this sensitive period (Bouchefra et al., 2022; Deary et al., 2007; Gottfredson, 1997). The neural basis of *Gf* has been attributed to a distinct, distributed network referred to as the parieto-frontal integration theory of intelligence, or the P-FIT network (Jung & Haier, 2007). Implicated brain regions within this classical model include the dorsolateral prefrontal cortex, superior and inferior parietal lobules, anterior cingulate cortex, and portions of the temporal and occipital lobes (Deary et al., 2010; Jung & Haier, 2007; Tschentscher & Sauseng, 2022; Wu et al., 2022). Notably, the developmental trajectory of *Gf* abilities aligns with changes in cortices implicated in the P-FIT model, which undergo significant structural and functional transformations throughout adolescence (Dumontheil, 2016; Estrada et al., 2019; Fuhrmann et al., 2020; Gur et al., 2021).

A recently growing body of work has identified the neural dynamics primarily supporting *Gf*. Specifically, theta, gamma, and alpha/beta oscillations from populations of neurons distributed throughout the P-FIT network have been consistently implicated in *Gf* development (Penhale et al., 2022; Taylor et al., 2020). These oscillatory responses have been shown to have a significant role in abstract reasoning performance, which is a fundamental element of *Gf* that enables the identification of patterns and relationships between multiple unfamiliar stimuli or scenarios to solve a problem. In adulthood, alpha and beta oscillatory dynamics are consistently reported during *Gf* processing, with some studies additionally reporting delta or theta responses underlying *Gf* (Arif et al., 2021; Ociepka et al., 2023; Penhale et al., 2022). These same patterns are noted in studies of children and adolescents, with theta dynamics being particularly sensitive to developmental changes (Heinrichs-Graham et al., 2022; Taylor et al., 2020; Ward et al., 2024). Further, sexual dimorphisms in developmental trends of neural activity have been demonstrated by extant *Gf* literature, particularly in the theta band (Taylor et al., 2020), despite similar behavioral performance between males and females. These previous studies investigating the neural dynamics underlying *Gf* development have all had relatively small study samples (less than 60 participants) for their analyses (Taylor et al., 2022a; Ward et al., 2024), and these smaller sample sizes restrict the statistical power of their findings. Our study aims to address this limitation and further characterize the development of neural dynamics supporting this critical cognitive construct.

The primary aim of this study lies in its replication of extant literature (i.e., Taylor et al., 2020) to further the characterization of fluid reasoning development that has been established, but in a larger, well-powered study sample. Therefore, the present study utilized the largest sample to date to investigate the oscillatory dynamics serving development of *Gf* abilities. Based on the results of previous studies, we hypothesized that youth would exhibit age-related increases in neural oscillatory responses (i.e., synchronizations of theta and gamma, desynchronizations of alpha and beta) within cortices implicated in the classical P-FIT model of intelligence (Basten et al., 2015; Jung & Haier, 2007; Kim et al., 2016), with distributed age effects anticipated in the dorsolateral prefrontal cortex, anterior cingulate cortex, inferior and superior parietal lobules, and inferior and superior frontal and temporal gyri. We also anticipated that age-related changes in neural dynamics would be coupled with better overall task performance (i.e., improved accuracy and reaction time). Additionally, we expected to detect some sexually dimorphic patterns of development in neural oscillatory responses; particularly, we anticipated differences primarily constrained to the theta band within superior and inferior frontal and temporal gyri, parietal cortices, and the dorsolateral prefrontal cortex (Taylor et al., 2020, 2022a). However, we did not predict significant differences in task performance between sexes, as extant literature has rarely reported such effects (Neubauer & Fink, 2003; Taylor et al., 2020).

## 2 Materials and Methods

### 2.1 Participants

We recruited 114 youth, ages 8–15 years (*M* = 11.55 ± 2.29 years; 57 males), from the greater metro area of Omaha, Nebraska, United States. All participants were typically developing, without any history of head trauma, neurological or psychiatric disorders, or other conditions affecting brain function. Participants were excluded according to general MEG/MRI exclusionary criteria such as the presence of metal implants, dental braces, permanent retainers, and/or any type of nonremovable ferromagnetic devices. Inclusion/exclusion criteria were confirmed by the parent. After a complete description of the study, written informed consent was obtained from the parent, and child participants provided assent. All procedures were approved by the local institutional review board. Methods for preprocessing and analysis follow Taylor et al. (2020), although the participants who took part in this study are a separate cohort.

### 2.2 Cognitive assessments

The two-scale Wechsler Abbreviated Scale of Intelligence (WASI-II; Wechsler, 2011) was administered by a trained research assistant in a quiet room. Raw scores were derived for the Matrix Reasoning and Vocabulary subtests in accordance with the assessment manual and used in further analyses. Matrix Reasoning subtest scores were used as a metric to determine fluid intelligence, and Vocabulary subtest scores were used to account for the effects of crystalized intelligence. Additionally, we computed each participant’s age- and sex-normed Full-Scale IQ Score (FSIQ-2) in accordance with the assessment manual to better characterize variability in intellectual capacity within the study sample. The WASI-II is known to have high reliability and validity in children and adolescents (Irby & Floyd, 2013; McCrimmon & Smith, 2013).

### 2.3 MEG experimental paradigm

**Fig. 1.**
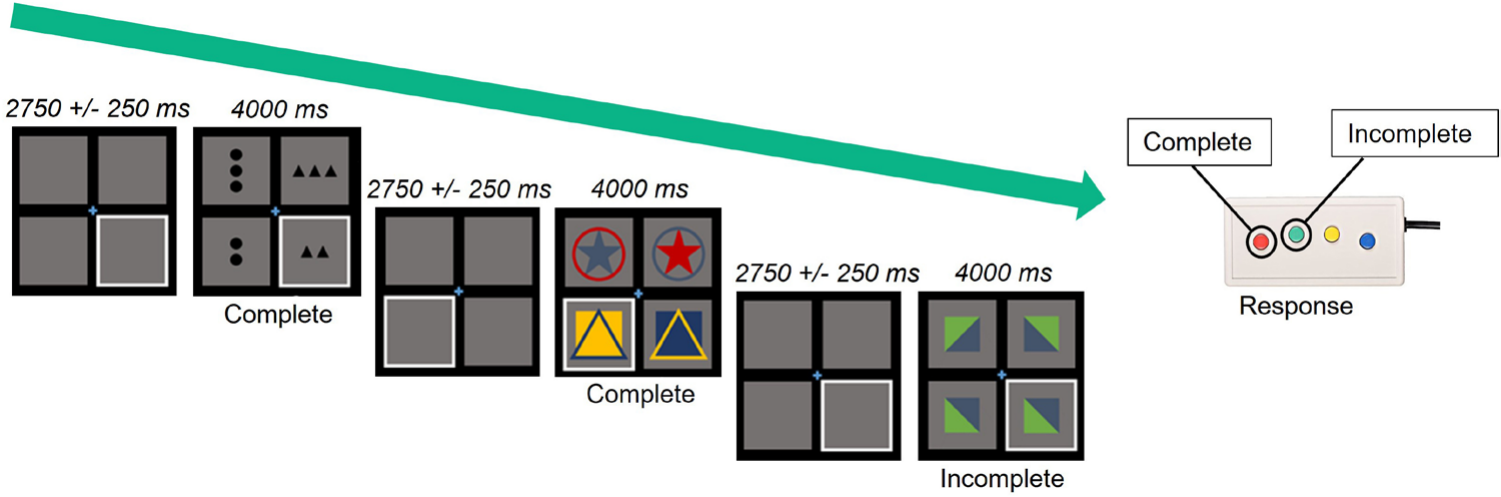
Abstract reasoning task paradigm. Participants were presented with a 2 × 2 grid of blank gray boxes, with a fixation cross appearing in the center of the grid. A white outline framed either the bottom left or right square during this time to indicate the location of the upcoming target stimulus. The grid was then populated with complex figures, at which time participants were asked to indicate with a button press whether the figure in the outlined square correctly completed the grid pattern.

Participants completed a custom, non-progressive abstract reasoning task that was adapted from the classic Raven’s Progressive Matrices during an MEG scan (Heinrichs-Graham et al., 2022; J. Raven, 2003; J. C. Raven, 1936; Taylor et al., 2020, 2022a; Ward et al., 2024). Participants were shown a centrally presented fixation cross in a 2 x 2 grid for a jittered period of 2,750 ms ± 250 ms. Either the bottom left or bottom right box was highlighted. An array of four complex figures was then presented for 4,000 ms. Participants were instructed to determine whether the complex figure in the highlighted box accurately completed the 2 x 2 grid given the pattern of images in the other three boxes (Fig. 1). Participants responded by pressing a button with their right index finger if the highlighted figure correctly completed the matrix, or by pressing a button with their right middle finger if the highlighted figure did *not* correctly complete the matrix. There was a total of 120 trials, equally split and pseudorandomized between correct and incorrect matrix completions. The task lasted approximately 14 min total with a 30-s break at the mid-point.

### 2.4 MEG data acquisition

Neuromagnetic responses were acquired within a two-layer magnetically shielded room using an MEGIN Triux Neo MEG system with 306 sensors (102 magnetometers and 204 planar gradiometers). Signals were sampled at 1 kHz with an acquisition bandwidth of 0.1–330 Hz. Each MEG dataset was corrected for head motion, and the signal space separation method with a temporal extension (tSSS; Taulu et al., 2005; Taulu & Simola, 2006) was applied for noise reduction. Briefly, preceding MEG recording, five coils were attached to the participant’s head and localized, together with the three fiducial points and scalp surface, with a 3D digitizer (Fastrak 3SF0002; Polhemus Navigator Sciences, Colchester, VT). Once the participant was positioned for MEG recording, an electric current with a unique frequency label (i.e., 322 Hz) was fed to each of the coils. This induced a measurable magnetic field and allowed each coil to be localized in reference to the sensors throughout the recording session.

### 2.5 MEG coregistration and structural MRI processing

All MEG measurements were transformed into a common coordinate system using the five coil locations as head coordinates. This coordinate system was used to coregister MEG data to each participant’s individual structural T1-weighted MRI prior to source reconstruction in BESA MRI (v. 3; BESA GmbH, Gräfelfing, Germany). Structural MRIs were acquired using a Siemens Skyra 3T MRI scanner with a 32-channel head coil and an MP-RAGE sequence with the following parameters: TR = 2,400 ms; TE = 1.94 ms; flip angle = 8°; FOV = 256 mm; slice thickness = 1 mm (no gap); voxel size = 1 × 1 × 1 mm. Acquired images were aligned to the anterior and posterior commissure and transformed into standardized space. Finally, 4.0 × 4.0 × 4.0 mm functional images computed during source analysis (i.e., beamforming) were also transformed into standardized space in the same manner as the structural MRI volumes.

### 2.6 MEG time–frequency transformation and statistics

The continuous magnetic time series was divided into epochs (duration: 6,500 ms), beginning 2,500 ms prior to the onset of the matrix stimuli and extending 4,000 ms afterward. Only trials for which the participant correctly responded to the stimulus were analyzed further. The baseline period was defined as 1,800 to 800 ms prior to the onset of the matrix stimuli to minimize any anticipation effects. Signal-space projection (SSP) was used to remove cardiac and eye blink artifacts across all MEG channels for each participant, and this was accounted for during source reconstruction (Uusitalo & Ilmoniemi, 1997). Artifactual epochs (those containing muscle artifacts, eye blinks/saccades, coughing, swallowing, etc.) were rejected based on a fixed-threshold method that was supplemented with visual inspection. Amplitude and gradient distributions per person were computed using all correct trials, and the trials with the highest amplitude and/or gradient values relative to the total distribution were excluded. Of note, individual signal amplitude (*M* = 1,190.63 ± 462.41 fT) and gradient (*M *= 39.19 ± 23.89 fT/s) thresholds were set for each participant due to differences among individuals in sensor proximity and head size, which strongly affect MEG signal amplitude. From the total correct trials (*M* = 96.65 ± 10.88), an average of 79.09 ± 13.32 (43 to 107) correct trials per participant remained for further analysis after artifact rejection. Using one-way ANCOVA, we assessed whether the number of trials covaried with age, sex, or their interaction. We found a significant main effect of age on the number of trials, with older participants typically having more trials than younger participants (*F*(1, 100) = 26.56, *p* < .001, η_p_^2^ = .21). There were no other significant main effects or interactions. Due to the potential confounding effect of the number of trials on age-related effects of interest, signal-to-noise ratio (SNR) was computed as the square-root of the number of included trials per person (Gross et al., 2013; Junghöfer et al., 2000; Vincent, 1992) and was included as a covariate of no interest in all analyses including MEG data (Pulliam et al., 2024).

Following artifact rejection, complex demodulation was used to transform all remaining epochs into the time–frequency domain (resolution: 2.0 Hz, 25 ms). The resulting spectral power estimations (per sensor) were averaged over trials and then normalized using the respective bin’s baseline power, which was defined as the mean power during the -1,800 to -800 ms window. Statistical analysis of the sensor-level spectrograms across all trials and gradiometers was used to determine the precise time–frequency windows used for source imaging. This analysis was limited to the first 1,000 ms following stimulus onset, which was selected to maximize focus on the abstract reasoning components, while minimizing the impact of other neural responses (e.g., motor) occurring later in each trial. The sensor-level statistical analysis followed an established two-stage procedure that was designed to minimize the risk of false-positive results while also maintaining reasonable sensitivity (see McDermott et al., 2017; Proskovec et al., 2019; Wiesman et al., 2017). Briefly, two-tailed paired-sample *t*-tests versus baseline were performed on each data point per spectrogram, and the resulting *t*-values were thresholded at *p* < .05. The time–frequency bins that survived the threshold were clustered with temporally and/or spectrally neighboring bins that also exceeded the threshold. A distribution of cluster values was then derived using nonparametric permutation testing and the significance level of the observed clusters was tested directly using this distribution (Ernst, 2004; Maris & Oostenveld, 2007). At least 1,000 permutations were computed to build a distribution of cluster values for each comparison. The significant time–frequency windows that were identified through this analysis were subjected to the beamforming analysis (see “Sensor-Level Results” in the Results section).

### 2.7 MEG source imaging and statistics

An extension of the linearly constrained minimum variance vector beamformer (Gross et al., 2001; Hillebrand et al., 2005; Veen et al., 1997), called Dynamic Imaging of Coherent Sources (DICS; Gross et al., 2001), was used to image cortical activity. This approach uses spatial filters in the frequency domain to estimate source power across the whole brain volume. Single images were computed from cross-spectral densities using all possible combinations of gradiometers averaged over the specified time-–frequency window, and the solution of the forward problem for each location on a grid specified by input voxel space. We computed noise-normalized source power per voxel in each participant using passive (i.e., baseline) and active (i.e., task) periods of equal duration and bandwidth (Hillebrand et al., 2005). The resultant images are referred to as pseudo-t maps, with units reflecting noise-normalized power differences between active and passive periods per voxel. MEG preprocessing and imaging were completed using BESA (V 7.1) software. Using the statistically selected time–frequency bands (see below), normalized differential source power was computed over the entire brain volume per participant (4.0 x 4.0 x 4.0 mm). The resulting 3D volumes of neural activity were averaged across all participants to assess the neuroanatomical distribution of each significant oscillatory response that was identified through the sensor-level analysis. Whole-brain multiple regressions were then computed with age, sex, and their interaction, along with SNR, as predictor terms for each map of significant oscillatory activity to examine developmental changes in the neural responses. All maps were threshold at a significance level of *p* < .005 and corrected for multiple comparisons using a cluster criterion (≥5 contiguous voxels), which was a conservative estimate based on the spatial smoothness of the image. Regressions were computed using SPM12.

Finally, we conducted follow-up analyses to examine whether the neural oscillatory activity identified in our developmental analyses related to behavioral performance on the task. Specifically, we examined whether oscillatory activity measured at the peak of each identified cluster mediated the relationship between the predictor of interest (i.e., age, sex, or their interaction) and task performance (i.e., reaction time and accuracy) on the abstract reasoning MEG task and the WASI-II matrix reasoning and vocabulary subtests. Because traditional tests of indirect effects (e.g., the Sobel test) often violate the assumption of normality, we utilized asymmetrical confidence intervals which best represent the true distribution of the indirect effect (i.e., the product of coefficients from the “a” and “b” paths). Thus, we examined the 95% confidence intervals of bias-corrected bootstrapped confidence intervals based on 1,000 bootstrapped samples, Efron and Tibshirani (1986), which provide a robust estimate of mediation effects and are asymmetrical (Fritz & MacKinnon, 2007). Mediation analyses were conducted in JASP 0.19.0.0.

## 3 Results

### 3.1 Demographic data and behavioral results

Of the 114 participants who completed the abstract reasoning task, 8 participants were excluded for task accuracy below 60%, and an additional 2 participants were excluded for excessively noisy or artifactual data collected during MEG. Therefore, the final sample consisted of 104 youth (*M* = 12.34 ± 2.23 years; 53 males). Average accuracy on the task was 80.37 ± 8.92%, and average reaction times were 2,052.62 ± 306.20 ms (1,104.39 to 2,707.85 ms). Accuracy and reaction time were significantly correlated, suggesting that youth who were more accurate on the task also tended to respond more quickly (*r* = -.51, *p* < .001). Derived from the WASI-II matrix reasoning (raw *M* = 19.51 ± 3.98) and vocabulary (raw *M* = 34.60 ± 6.54) subtests, the average normed FSIQ-2 score was 111.87 ± 13.26 (78 to 150).

We computed separate one-way ANCOVAs to assess relationships between task performance (i.e., reaction time or accuracy) and age, sex, and their interaction. We found a significant main effect of age on accuracy (*F*(1, 100) = 45.79, *p* < .001, η_p_^2^ = .31), such that as age increased, performance on the task improved (b = 2.21, *p* < .001). There was also a main effect of sex on accuracy (*F*(1, 100) = 3.96, *p* = .049, η_p_^2^ = .04), wherein males displayed marginally higher scores than females on the task (b = 2.88, *p* = .049). Additionally, reaction time decreased as a function of age (*F*(1, 100) = 35.45, *p* < .001, η_p_^2^ = .26), as older youth completed the task more quickly (b = -80.37, *p* < .001). There was no significant effect of the interaction between age and sex on measures of task performance.

### 3.2 Sensor-level results

**Fig. 2.**
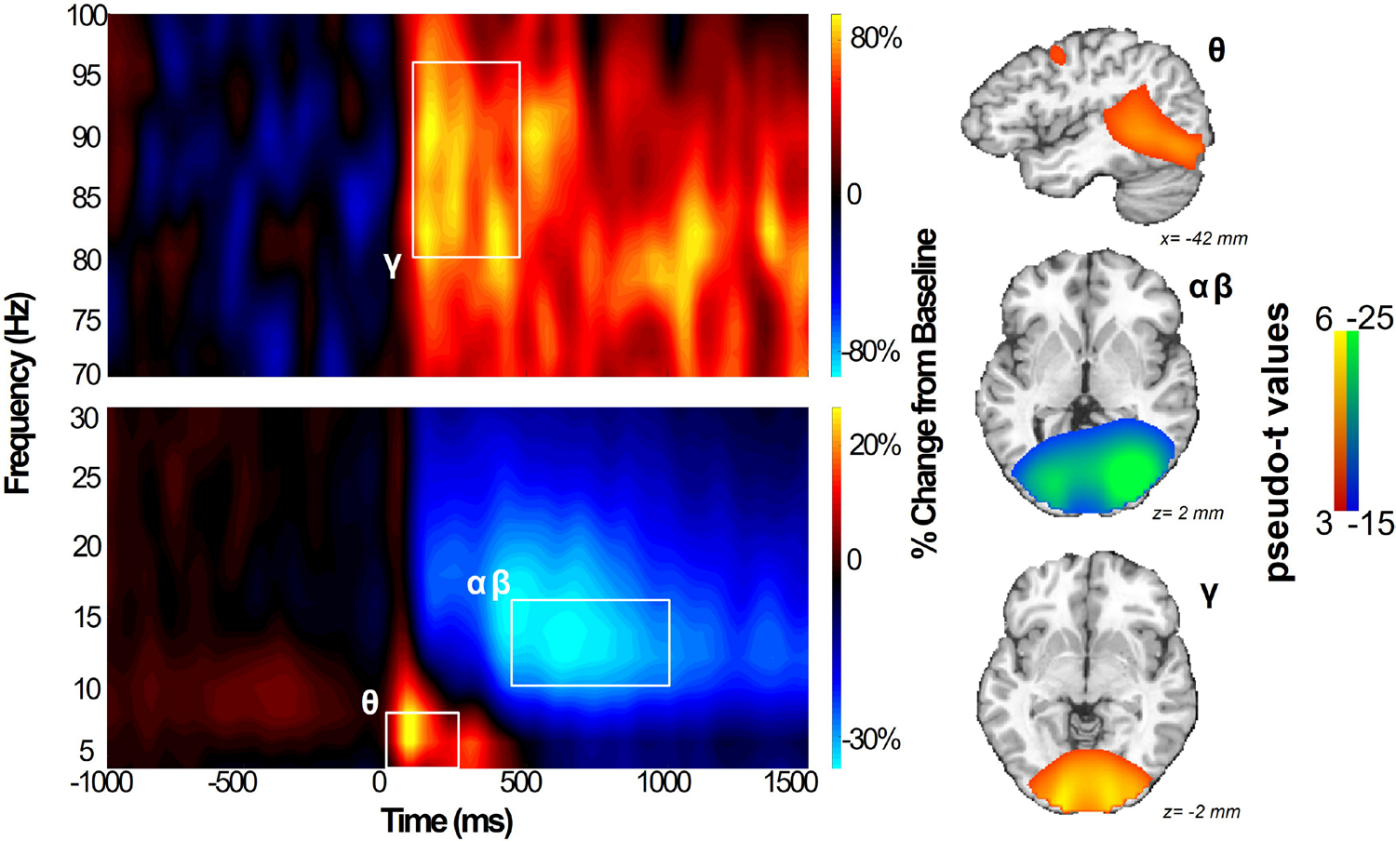
Spectrograms of significant neural oscillatory responses across trials of abstract reasoning task. We conducted time–frequency decomposition and permutation-corrected statistical analysis (*p* < .05, corrected). Resulting spectrograms of MEG sensors indicated three time–frequency bins of interest during the 1,000 ms directly following stimulus presentation. Activity in order of appearance from lowest to highest frequency: theta (4–8 Hz, 0–250 ms), alpha/beta (10–16 Hz, 450–1,000 ms), and gamma (80–96 Hz, 100–475 ms). All gradiometers were included for statistical analysis, with the sensor presenting the clearest responses across all bins (MEG2043) displayed here. Whole-brain average maps showing source reconstructions of oscillatory responses are displayed for each imaged time–frequency bin, with pseudo-t values spanning 3 to 6 in theta and gamma bands and -15 to -25 in the alpha/beta band.

Statistical analysis of the time–frequency spectrograms showed a significant increase in theta power from 4 to 8 Hz spanning posterior, central, and frontal sensors from 0 to 250 ms. Further, there was a decrease in alpha/beta power between 450 and 1,000 ms from 10 to 16 Hz, and an increase in gamma between 100 and 475 ms from 80 to 96 Hz localized to more posterior sensors (*p’*s < 0.001) (Fig. 2).

### 3.3 Source-level results

Time–frequency spectrograms were imaged with a beamformer for each participant to identify the specific brain regions that generated the oscillations identified at the sensor level. The consequent images were then assessed for outlier exclusions based on artifactual data, with exclusion criteria including oscillatory activity bleeding across bands and non-physiological artifactual activity in individual bands that did not manifest across oscillatory bands within a participant. Hence we did not exclude participants entirely based on artifactual data from one band. Afterward, 91 participants remained with viable data in the theta band, 97 in alpha/beta, and 94 in gamma. The functional maps resulting from this imaging were averaged for all participants (Fig. 2). Early oscillatory activity was observed across trials in the theta band (4–8 Hz) from 0 to 250 ms after stimulus onset in frontoparietal and occipital cortices. In addition, strong gamma activity (80–96 Hz) was seen in the occipital region from 100 to 475 ms. Later activity in the alpha/beta band (10–16 Hz) was seen in the parietal and occipital cortices from 450 to 1,000 ms. To determine the effects of age, sex, and their interaction on the neural dynamics serving abstract reasoning, beamformed maps of significant oscillatory responses were submitted to whole-brain regressions with age, sex, and their interaction as predictors of interest. All analyses controlled for SNR as a covariate of no interest.

### 3.4 Main effects of age

**Fig. 3.**
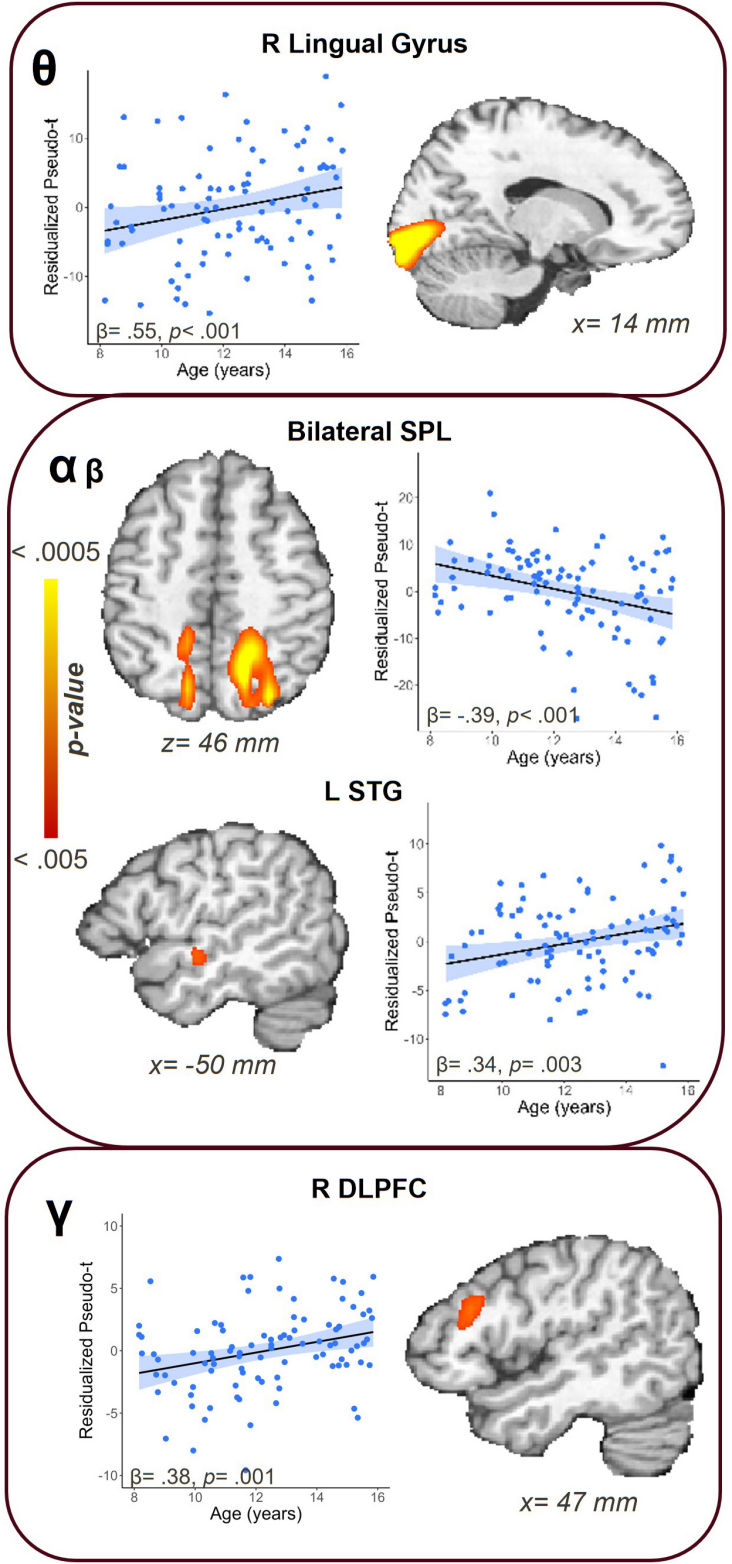
Main effects of age on neural dynamics during the abstract reasoning task. Scatterplots show the residualized pseudo-t values after controlling for sex and SNR. Statistical *F* maps show clusters for which age was significantly related to oscillatory responses. Stronger age-related theta synchronization was located in the right lingual gyrus, while gamma activity showed stronger synchronization in the right dorsolateral prefrontal cortex (DLPFC) as a function of age. In the alpha/beta window, stronger desynchronization responses appeared with age in the bilateral superior parietal lobules (SPL). In the left superior temporal gyrus (STG), alpha/beta desynchronizations weakened as a function of age.

We detected multiple main effects of age on neural dynamics during the task (Fig. 3). Theta responses tended to become significantly stronger (i.e., more synchronized) with increasing age in the right lingual gyrus (β = .55, *p* < .001). In the alpha/beta band, we saw significantly stronger responses (i.e., greater desynchronization) as a function of age in the bilateral superior parietal lobules (SPL; βs = -.33 and -.39, *p* = .002 and *p* < .001) and the right superior insula (β = -.32, *p* = .004), coupled with age-related decreases in responses (i.e., less desynchronization) in the left superior temporal gyrus (STG; β = .34, *p* = .003). Finally, in the gamma band, we noted increases in power (i.e., stronger synchronization) as a function of age in the bilateral SPL (β’s = .38 and .45, *p* = .001 and *p* < .001) and the right dorsolateral prefrontal cortex (DLPFC; β = .38, *p* = .001).

Of note, we recognize the potential for contamination from early/preparatory motor responses, particularly in the alpha/beta responses for which our beamformed window extends the latest. To investigate this, we first computed partial correlations between the extracted pseud-t values for each of the identified alpha/beta responses and average reaction times, covarying out age, sex, and SNR. Reaction times were positive correlated with alpha/beta responses in the right and left SPL such that children with faster average response times tended to have stronger neural responses (i.e., larger desynchronizations; *r’*s = .305 and .267, *p’*s = .003 and .010, respectively).

Next, we determined the effect of accounting for reaction times on the identified age relationships from these two clusters. We ran our regressions with age, sex, SNR, and reaction time all as predictors of alpha/beta responses extracted from the right and left SPL, and we compared the regression parameter estimates (unstandardized betas) between the models with and without reaction time as a predictor (i.e., our originally reported models versus updated models accounting for reaction time). Models were computed as hierarchical linear regressions (Type II sums of squares), changing the order of entry for age and reaction time in separate models. This allowed us to evaluate the change in *R*^2^ following the inclusion of age and reaction time to the model, thus ensuring that the order of predictor entry did not affect parameter estimates. We then computed a Z score to compare the age parameters from the models with versus without reaction time included as follows:

$$Z=\;\frac{\left(b_{1}-\;b_{2}\right)}{\sqrt{SE_{1}^{2}+\;SE_{2}^{2}}}.$$

Regardless of the order of entry, both reaction time and age were uniquely significant predictors of variance in pseudo-t values. In the final models including both variables, although the age effects in the expanded models were slightly weaker for both the right (b_without RT_ = -1.701, SE = 0.454; b_including RT_ = -1.249, SE = 0.475) and left SPL (b_without RT_ = -1.418, SE = 0.457; b_including RT_ = -1.021, SE = 0.483), neither estimate was statistically significantly different after accounting for reaction times (Z_right_ = 0.688, *p* = .49; Z_left_ = 0.597, *p* = .55).

### 3.5 Main effects of sex

**Fig. 4.**
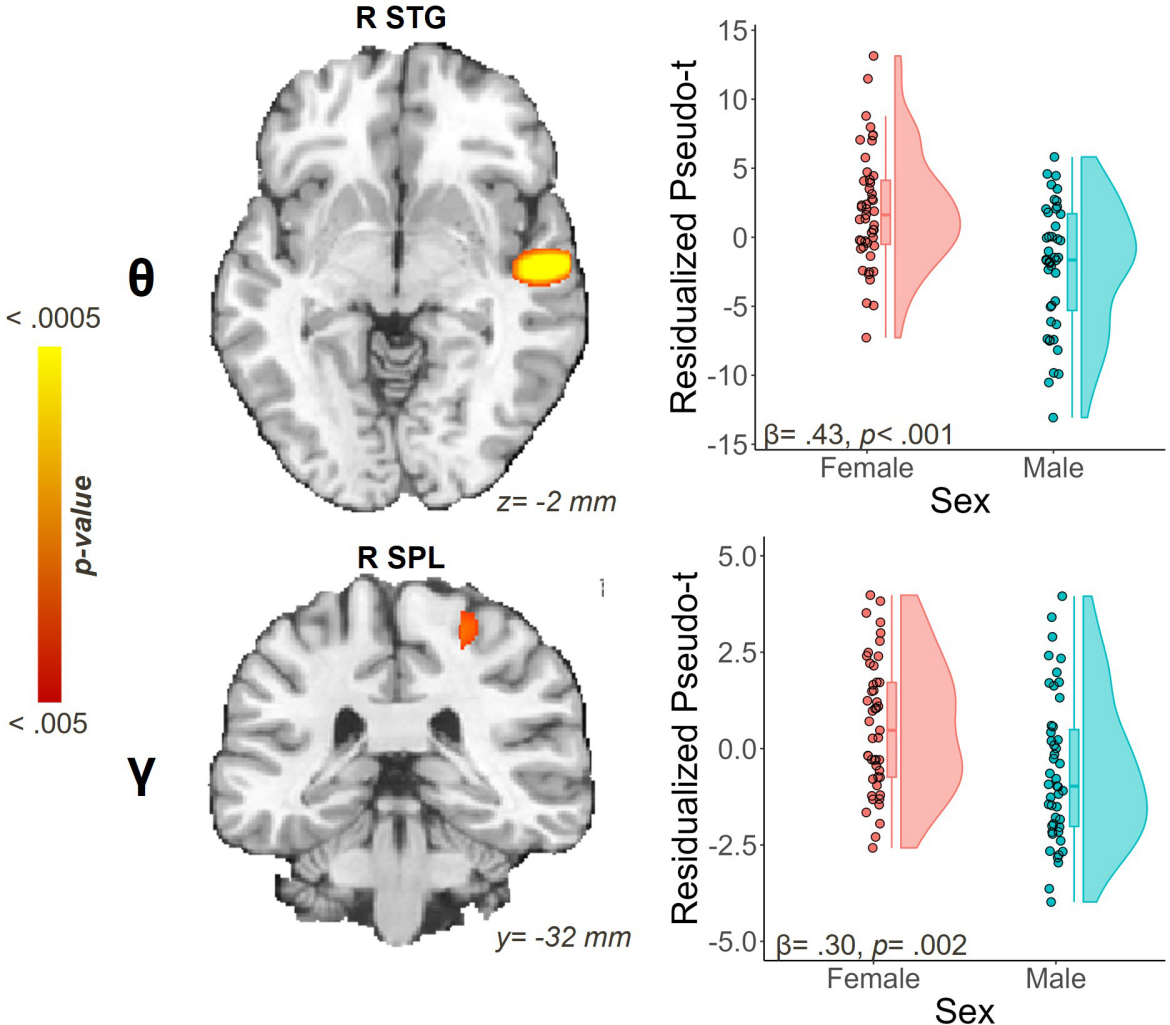
Main effects of sex impacting neural dynamics during the abstract reasoning task. Raincloud plots show the residualized pseudo-t values after controlling for age and SNR. Statistical *F* maps show right-hemispheric clusters for which biological sex was significantly related to oscillatory responses. Stronger theta synchrony was concentrated in the superior temporal gyrus (STG) and stronger gamma synchrony in the superior parietal lobule (SPL) for female participants.

We identified two main effects of sex on oscillatory activity during the task (Fig. 4). We observed significantly stronger responses within the theta band for females than for males in the right STG (β = .43, *p* < .001). Likewise, gamma responses in the right SPL tended to be significantly stronger among females relative to males (β = .30, *p* = .002).

### 3.6 Interaction between age and sex

**Fig. 5.**
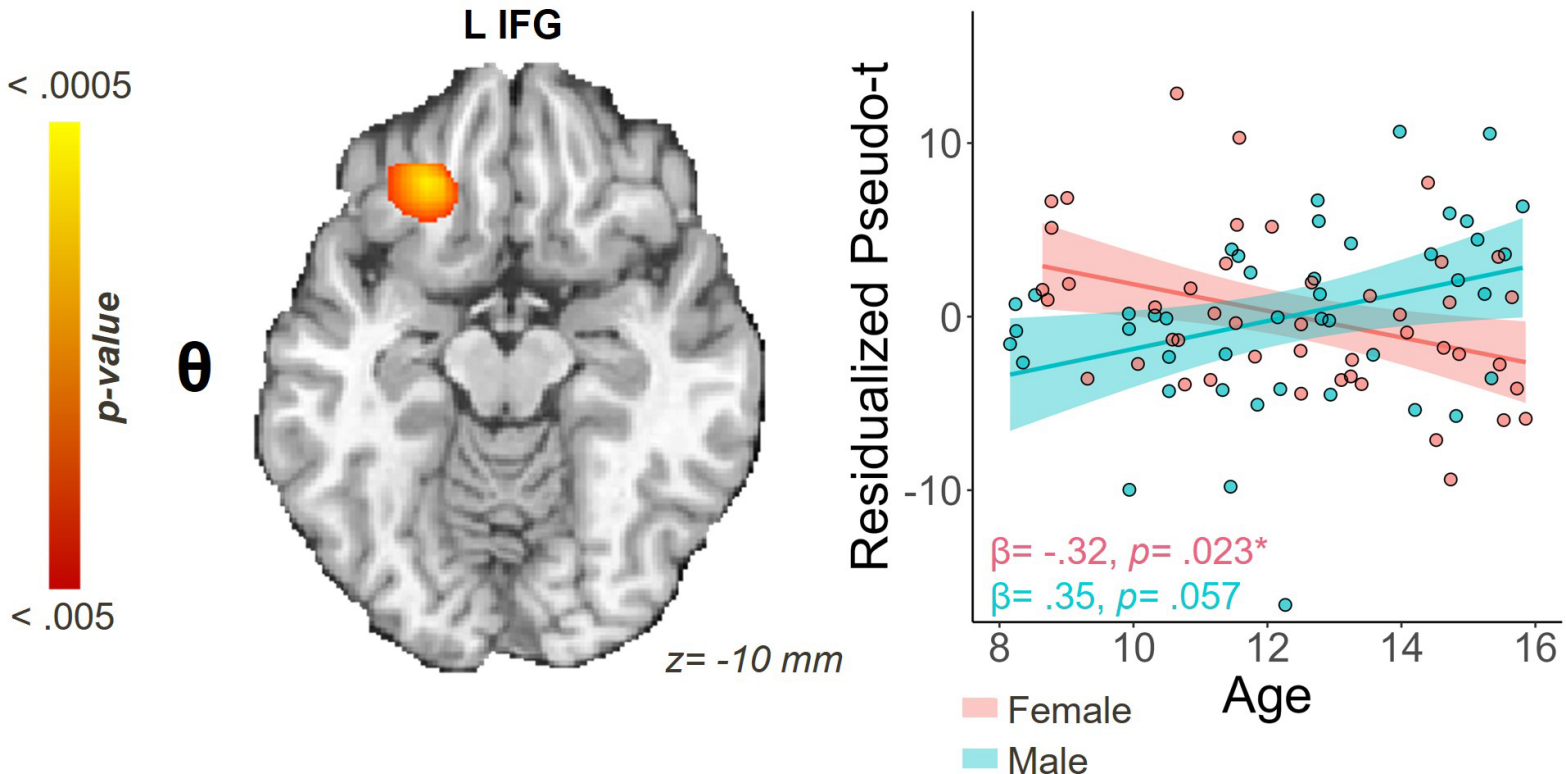
Age-by-sex interaction within the theta band. Scatterplot shows the residualized pseudo-t values after controlling for SNR. Statistical *F* map shows a significant cluster in the left inferior frontal gyrus (IFG) for which the interaction of age and sex was significantly related to oscillatory responses.

We identified one region for which there was a significant age-by-sex interaction (β = -.49, *p* < .001) on neural dynamics serving fluid reasoning (Fig. 5). In the theta band, we saw a stronger response (i.e., greater synchronization) among younger females that gradually became weaker with age in the left inferior frontal gyrus (IFG; β = -.32, *p* = .023).

### 3.7 Relationship with behavior

**Fig. 6.**
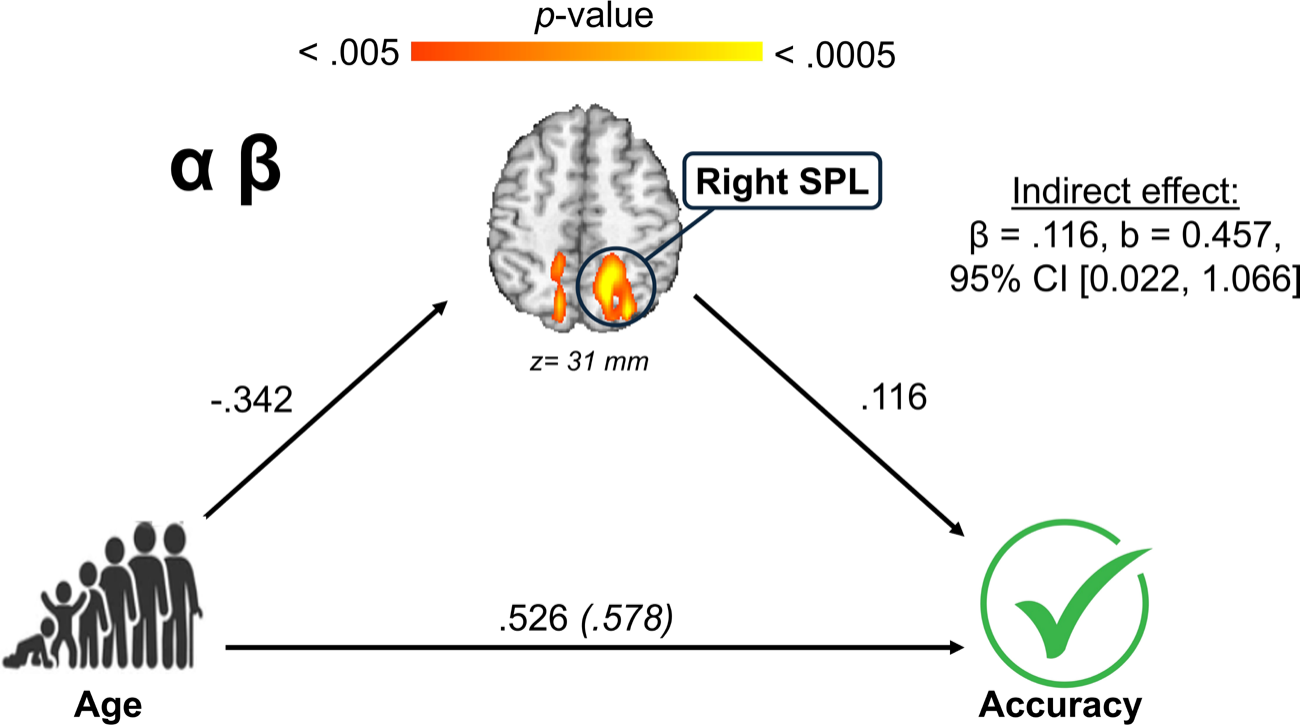
Age-related alpha/beta desynchronization in the right superior parietal lobule (SPL) mediated the relationship between age and accuracy on the abstract reasoning MEG task. The mediation figure shows the standardized regression coefficients between age and alpha/beta activity in the right SPL, and between this age effect in the right SPL and accuracy, as well as the total and direct effects of age on accuracy.

**Fig. 7.**
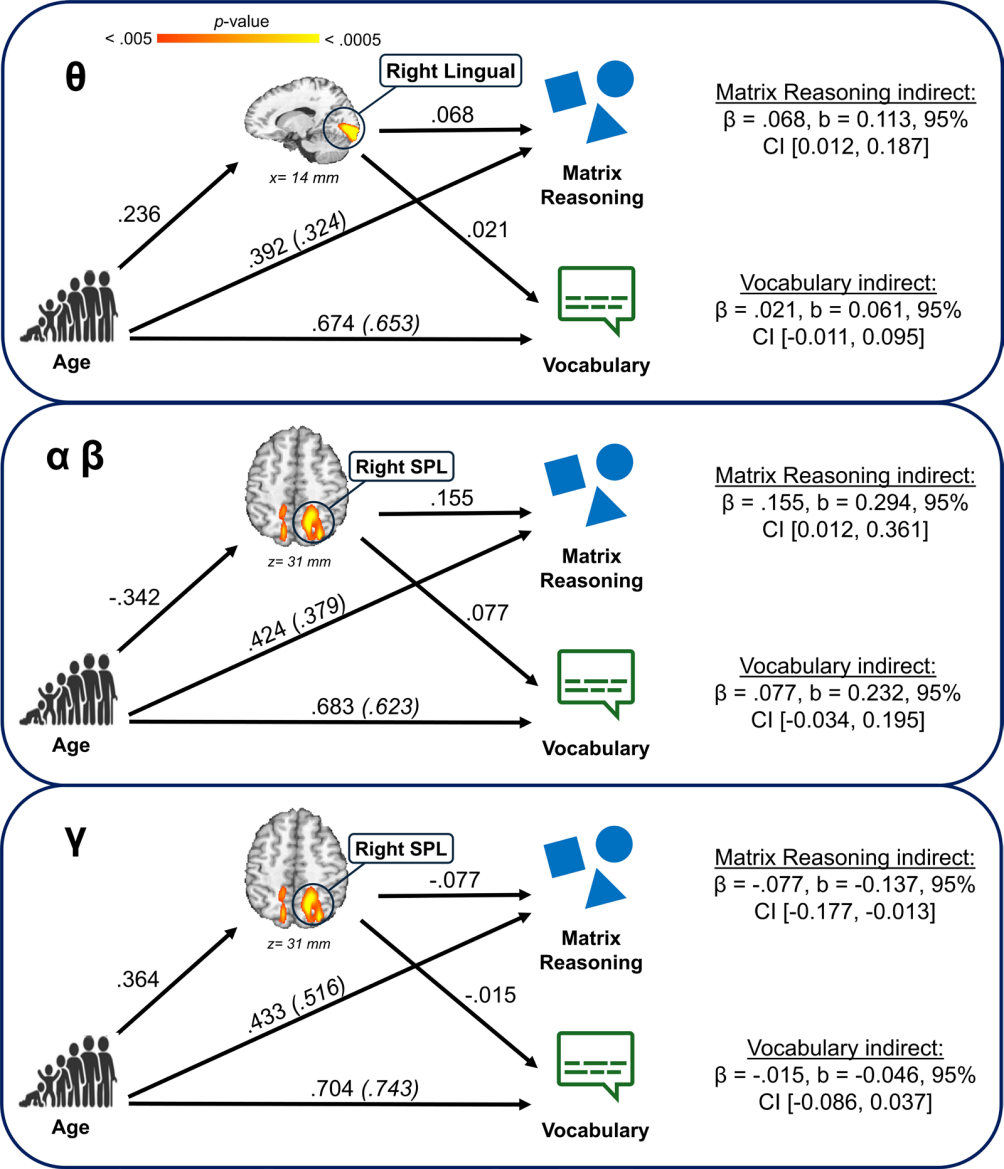
Neural oscillatory activity mediated the relationship between age Matrix Reasoning scores, but not vocabulary scores from the WASI-II. All parameters listed on the models are standardized coefficients. Indirect effects are listed with each model for both the matrix reasoning and vocabulary score outcome variables. Note: “SPL” = superior parietal lobule.

We conducted a set of mediation analyses to investigate the degree to which neural dynamics in the examined clusters of interest mediated the relationship between our primary predictors (i.e., age, sex, or their interaction) and either performance during the MEG abstract reasoning task (i.e., reaction time and accuracy), or scores on the matrix reasoning and vocabulary scales from the WASI-II. The analyses yielded several significant indirect effects wherein activity in the observed clusters mediated the relationship between age and behavioral metrics. Alpha/beta activity in the right SPL mediated the relationship between age and accuracy during the MEG task (Fig. 6). Specifically, older children had stronger alpha/beta oscillatory responses in the right SPL, which were associated with higher mean accuracy on the abstract reasoning task (β = .116, b = 0.457, 95% CI [0.022, 1.066]). Furthermore, we noted several instances for which age-related changes in neural dynamics mediated the links between age and performance on the WASI-II matrix reasoning scale (Fig. 7). We found that older children had stronger theta responses in the right lingual gyrus, which were then associated with higher scores on the matrix reasoning subtest (β = .068, b = 0.113, 95% CI [0.012, 0.187]). Older children similarly tended to display stronger alpha/beta responses in the right SPL, which were associated with higher matrix reasoning scores (β = .155, b = 0.294, 95% CI [0.012, 0.361]). Older children additionally had stronger gamma responses in the right SPL, and this was linked with lower matrix reasoning scores (β = -.077, b = -0.137, 95% CI [-0.177, -0.013]). There were no instances for which vocabulary scores were significantly mediated by the effects of age, sex, or their interaction.

## 4 Discussion

The present study sought to characterize the development of the neural dynamics serving fluid reasoning in a large sample of children and adolescents. As expected, we identified age-related improvements in task performance for both the WASI-II matrix reasoning subtest and the MEG abstract reasoning task. With respect to neural dynamics, we identified oscillatory responses in the theta, alpha/beta, and gamma frequencies distributed throughout key regions implicated in the P-FIT model, each of which showed sensitivity to age and/or sex. Perhaps our most interesting finding was that age-related changes in these multispectral responses were robustly coupled with performance on the abstract reasoning task and the matrix reasoning subtest. We discuss our novel findings in detail below.

We found several instances for which oscillatory dynamics mediated the relationship between age and behavioral outcomes. Age-related increases in theta responses in the right lingual gyrus were related to higher scores on the WASI-II matrix reasoning subtest, which resembled findings in the visual cortex by prior *Gf* studies (Doucet et al., 2022; Yang et al., 2019). The lingual gyrus is implicated in visual memory and imagery processes (Olivetti Belardinelli et al., 2009; Roland & Gulyás, 1995; Shi et al., 2019), and its successful development is essential in supporting downstream processing of complex stimuli. Such visual attention processes often exhibit patterns of improvement over time as youth develop (Amso & Scerif, 2015; Wass et al., 2025), which could reasonably lend to better performance on this attentionally demanding subtest. Another key mediation finding was that older children displayed stronger alpha/beta oscillatory responses in the right SPL, which was related to higher accuracy on both the abstract reasoning MEG task and on the WASI-II matrix reasoning subtest. This aligns with our understanding of the SPL, which is implicated in the P-FIT model and acts as a hub of visual–spatial perception, mental imagery, and attention (Basten et al., 2015; Coull & Frith, 1998; Jung & Haier, 2007; Malhotra et al., 2009; Seydell-Greenwald et al., 2017). Converse to this alpha/beta SPL finding, older youth also showed stronger *gamma* responses in the right SPL, which were associated with *lower* scores on the matrix reasoning task. Although the SPL is implicated in the P-FIT model of intelligence, this region has additionally been associated with impairments in abstract reasoning following bilateral stimulation (Tsujii et al., 2011). Overall, distinct frontoparietal connections appear to support fluid reasoning most effectively at various stages of adolescent development (Wendelken et al., 2016). Importantly, we did not detect any significant relationships between developmentally sensitive neural dynamics and scores on the vocabulary subtest of the WASI-II, though several showed robust links to matrix reasoning scores. These findings may denote a degree of specificity to fluid reasoning abilities. However, not all clusters showed significant behavioral effects. It is possible that the age- and sex-related changes in neural dynamics in other clusters may be indicative of broader, more generalized neurocognitive development. Further work is needed to more precisely determine what cognitive constructs are supported by the maturation of these neural dynamics.

We noted multiple other instances for which multispectral dynamics exhibited developmental sensitivity in brain regions implicated within the P-FIT model of intelligence (Jung & Haier, 2007), including in the DLPFC and STG. In the right DLPFC, gamma activity tended to become stronger as age increased, which aligns with prior literature concerning the right DLPFC’s role in the development of intelligence (Arif et al., 2021; Barbey et al., 2013; Santarnecchi et al., 2021), goal-driven attention and problem-solving (Jones & Graff-Radford, 2021; Tomasino & Fabbro, 2016) and working memory capacity (Engle et al., 1999; Miller & Cohen, 2001; Stocco et al., 2021). With respect to alpha/beta oscillations, age-related increases in responses in the right superior insula could reflect a heightened capacity to manage cognitive load during the abstract reasoning task, as this region is particularly active during complex tasks with greater cognitive demands (Gogolla, 2017; Uddin et al., 2017). Notably, similar developmental results have been detected within these regions and oscillatory bands that specifically draw on alternative cognitive demands (i.e., attention, working memory) (Petersen & Posner, 2012; Posner, 1980; Son et al., 2023; M. Wang et al., 2020; Zhu et al., 2010). Conversely, alpha/beta oscillations in the left STG exhibited a trend of weakening as a function of age, which may hearken to the STG’s role as a primary hub in the P-FIT model. Particularly, the left STG is implicated in increasing efficiency of *Gf* abilities (Taylor et al., 2022a). Curiously, this finding contradicted the reported outcomes of a previous longitudinal study from Taylor et al. (2022a), which utilized the same abstract reasoning MEG task used in the present study. The study reported increases in beta responses within the left STG within participants 1 year after their initial MEG scan. However, within- and between-person findings can be discrepant (Curran & Bauer, 2011; Kievit et al., 2013; Taylor et al., 2022b), so it may not be surprising that the associations reported by the Taylor et al. (2022a) longitudinal study differ from our cross-sectional results. Broadly, our multispectral findings indicated that the transition from middle childhood into adolescence is a sensitive period of maturation for higher-order cognitive processes and their underlying neural structures.

In addition to our developmental findings, we found that female participants displayed stronger theta activity in the right STG and gamma activity in the right SPL. The right STG has a key role in stimulus-centered spatial processing and visual search processes (Gharabaghi et al., 2006; Shah-Basak et al., 2018), while the right SPL has a distinct role in visual attention and reasoning (Wang et al., 2014). In addition, we detected one sexually divergent developmental effect, wherein female youth tended to show an initially stronger theta response in the left IFG than their male counterparts, which decreased as a function of age. Prior research has found that females tend to exhibit signs of neural maturation earlier than their male counterparts during adolescence, often showing neural markers of development several years earlier (Giedd et al., 2012; Lenroot & Giedd, 2010). Hence, this decrease in oscillatory activity may reflect the dimorphic developmental stages between male and female youth. In another study, Taylor et al. (2020) directly linked oscillatory activity in the left IFG among females to *Gf* development while utilizing the same abstract reasoning task implemented in the present study. Thus, the decrease we detected in theta activity with age in the left IFG for female youth was corroborated by extant literature employing the same task in a smaller sample, which gave weight to the replicability and robustness of the finding.

Intriguingly, the majority of the neural findings identified were lateralized to the right hemisphere, despite prior developmental *Gf* research reporting relatively distributed, bilateral effects of age, sex, and their interaction (Taylor et al., 2020, 2022a; Wright et al., 2008). This trend of right-lateralized effects comes as an even greater surprise when considering that the P-FIT network tends to be described as a more left hemispheric network, although it does span bilaterally (Jung & Haier, 2007). It could be that left hemispheric processes mature at a different pace than those localized to the right hemisphere (Liang et al., 2025; Nagel et al., 2013; Thatcher et al., 1987), or that we simply did not harness these nodes as strongly with the current task design. There are a multitude of alternative methods to measure fluid reasoning (Cattell, 2013; Kaufman & Kaufman, 2016; Lee et al., 2021; Schrank & Wendling, 2018), which may better elucidate the maturational trajectory of left-localized neural dynamics. Alternatively, it is possible that there was more variability in the nature of development in our larger sample such that the relationships between age and neural oscillatory activity were less consistent when explored in a greater, more generalizable sample, which adds credence to the need for this replicative work.

It is essential to acknowledge several limitations of this study. First, this study employed a cross-sectional design, which may not sufficiently capture the scope of *Gf* development. Future studies may elect to remedy this by including a longitudinal extension to the study. Additionally, we solely considered chronological age as a measure of maturation, which neglects to consider the role of other markers of maturation, including pubertal development. Incorporating alternative metrics of maturation may further the elucidation of *Gf* abilities and the trajectories of its underlying neural substrates during childhood and adolescence. Neural oscillatory activity observed within this study may not exclusively capture fluid reasoning, but might reflect a range of domains including cognitive control and attention, as well as potential contamination from early/preparatory motor responses, particularly in the extended alpha/beta window. Conducting a trial-level analyses would better account for variability in response times and, thus, account for the role of motor responses. Additionally, single trial-level analyses could yield different patterns of oscillatory activity and characterize the strength of the links between neural activity and behavior. Thus, future work may benefit from utilizing a trial-by-trial approach both to account for preparatory motor activity and to assess the unique aspects of development that are driven by the manner of processing that occurs for each trial type. Another limitation is that the current study did not explicitly explore both periodic and aperiodic neural dynamics. Future analyses regarding non-oscillatory (aperiodic) activity in children and adolescence would offer an intriguing opportunity to garner a deeper, more robust understanding of the developmental patterns identified in the current study. Finally, we examined an exclusively neurotypical sample, which does not represent the vast array of neurodevelopmental differences present in the wider population. Therefore, our findings may not effectively capture *Gf* development in populations with disabilities that affect higher-order cognition (i.e., attention-deficit hyperactivity disorder).

To conclude, the present study aimed to clarify the developmental trajectory of neural dynamics underpinning fluid intelligence (*Gf*) across childhood and adolescence in a large sample of over 100 youth. Our findings demonstrated that theta, alpha/beta, and gamma oscillatory bands are critically involved in the maturation of *Gf* during this formative period. We observed age-related improvements in task performance on the WASI-II matrix reasoning subtest and the MEG abstract reasoning task which were directly related to developmental changes in multispectral neural dynamics in the right SPL and right lingual gyrus. Additional age- and sex-related trends emerged that were distributed throughout the classical P-FIT network including the DLPFC, STG, and IFG, all of which have been critically implicated in *Gf* abilities across the lifespan. In replicating the extant literature, our study addressed the previous limitation of statistical power derived from small sample sizes. The findings of our large sample served to elucidate the neural substrates underpinning *Gf* and reinforce the importance of the P-FIT network in the development of *Gf*.

## Data Availability

The data reported herein will be made publicly available upon completion of the study via the Collaborative Informatics and Neuroimaging Suite (COINS; https://coins.trendscenter.org/).
